# Upregulation of TPX2 by STAT3: Identification of a Novel STAT3 Binding Site

**DOI:** 10.1371/journal.pone.0113096

**Published:** 2014-11-17

**Authors:** Rossana Cocchiola, Caterina Grillo, Fabio Altieri, Silvia Chichiarelli, Carlo Turano, Margherita Eufemi

**Affiliations:** 1 Department of Biochemical Sciences, Sapienza University of Rome, 00185 Rome, Italy; 2 Istituto Pasteur-Fondazione Cenci Bolognetti, Piazzale Aldo Moro 5, 00185 Roma, Italy; Institut de Génétique et Développement de Rennes, France

## Abstract

TPX2, a protein involved in mitosis, is considered a good marker for actively proliferating tissues, highly expressed in a number of cancer cells. We show the presence of high-affinity binding site for STAT3 in the 5′-flanking region of the *Tpx2* gene, which is *in vivo* bound by activated STAT3. A specific STAT3 peptide inhibitor represses the expression of the *Tpx2* gene and inhibits the binding of STAT3 to its consensus sequence in human cell lines where STAT3 is activated. These results indicate that activated STAT3 contributes to the over-expression of *Tpx2* through the binding to an enhancer site.

## Introduction

TPX2 is one of the several proteins involved in the complex process of mitosis, and has been identified as one of the microtubule-associated proteins. TPX2, also known as Dil-2, p100, HCA519 or C20orf1/2, has been initially described as a protein mediating the binding of the kinesin-like protein 2 (Xklp2) of Xenopus to microtubules. TPX2 is also necessary for the nucleation of microtubules around chromosomes, for spindle pole organization, for the activation of the mitotic kinase Aurora A and its targeting to spindle microtubules [1]. In interphase TPX2 shows a nuclear distribution but becomes localized to spindle poles during mitosis, through a functional interaction with the dynein-dynactin complex. Its gene is expressed during S, G2 and M phases of cell cycle, and the protein undergoes an extensive degradation after mitosis [2]. Due to these properties TPX2 has been considered a good histological marker for actively proliferating tissues, and hence for tumours, where it has been established that TPX2 expression is altered [3]. It is noteworthy that also one of its targets, i.e. the Aurora kinase A, is frequently overexpressed in tumours, indicating that both proteins are important for tumour formation or progression [4]. Little is known about the regulation of *Tpx2* gene transcription. An inspection of the 5′-region of human *Tpx2* gene on chromosome 20 revealed the presence of many consensus sequences for transcription factors, among which are some putative sites for STAT factors.

The STAT family members are latent transcription factors present in the cytoplasm that mediate cytokines or growth factors signalling and are involved in cell differentiation, proliferation and survival [5].

Following the binding of cytokines or growth factors to their receptors, STATs are activated by tyrosine phosphorylation which induces their dimerization, or a conformational change, followed by nuclear import and binding to their specific DNA-response elements. Some STATs, and particularly STAT3, not only fulfil their roles in normal cell signalling, but may contribute to oncogenesis. Bromberg et al. [6] have shown that a mutated form of STAT3, capable of a spontaneous dimerization and therefore being permanently activated, induces cellular transformation in immortalized fibroblasts. STAT3 is constitutively activated in all Src-transformed cells, and also in a variety of human cancers [7]–[8].

## Materials and Methods

### Cell Cultures

HeLa, HepG2 and A431 cells were obtained from ATCC. Human melanoma (M14) cells were a kind gift from Prof E. Agostinelli (Sapienza University, Rome) [9] and human thyroid anaplastic carcinoma (ARO) cells were a kind gift from Prof A. Fusco (IEOS-CNR of Naples) [10]. Cells were grown to 80% confluence at 37°C in 5% CO_2_ in RPMI 1640 medium, added with 1% sodium pyruvate, 10% fetal bovine serum, 2 mM glutamine, 100 µg/ml streptomycin, and 100 U/ml penicillin. M14 and ARO cells were treated for 24 h and 48 h with 1 mM phosphopeptide PpYLKTK-mts for inhibition experiments [11]–[12].

### Electrophoretic Mobility Shift Assay

**Figure 1:**
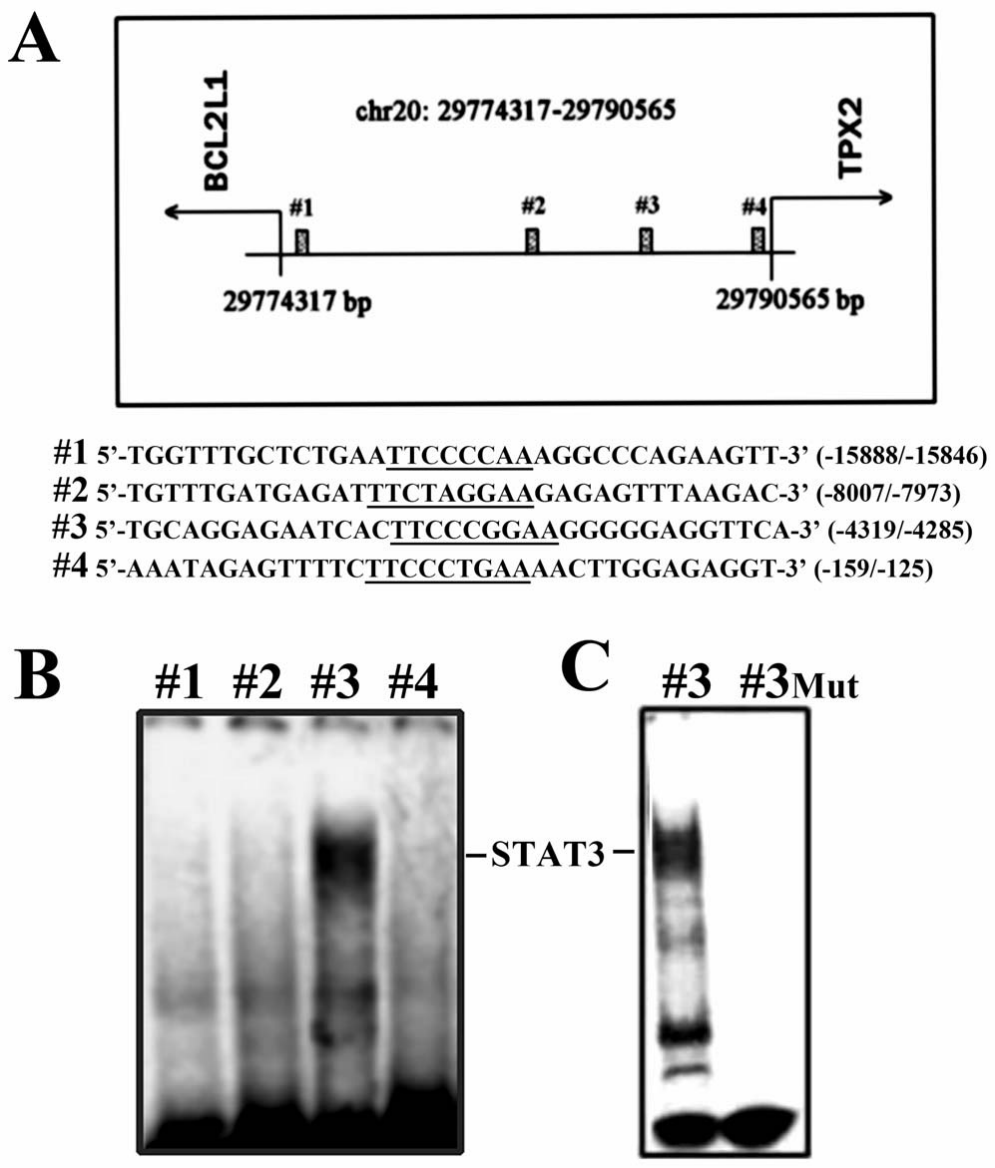
Regulation of the *Tpx2* promoter by STAT3. (**A**): Sequences and positions of putative STAT3-binding elements on the 5′-flanking region of the *Tpx2* gene. (**B**): EMSA experiment performed using, as probes, ^33^P-end labeled double-stranded oligonucleotides corresponding to the four potential STAT3-binding sites present in the region between the *Bcl2l1* and the *Tpx2* genes. (**C**): Effect of base mutations of the STAT3 consensus sequence. EMSA experiment carried out with mutated probe #3 (TGCAGGAGAATCACTTCC**AT**G**TT**GGGGGAGGTTCA, were the mutated bases are indicated in bold characters).

STAT3-DNA binding was analyzed by electrophoretic mobility shift assay (EMSA). EMSA experiments were performed using as probes ^33^P-end labeled double-stranded oligonucleotides, corresponding to the four potential STAT3-binding sites present in the region between the *Bcl2l1* and the *Tpx2* genes. The probes, showed in figure 1A, were incubated 30 min at room temperature with 10 µg of nuclear extracts prepared from M14 and ARO cells. Nuclear extracts were obtained from nuclei purified as previously described in Eufemi at al. [13]. Nuclei were resuspended twice in a hypertonic buffer (20 mM Hepes ph 8, 20% v/v glycerol, 0.4 M KCl, 1 mM EDTA, 1 mM DTT), supplemented with proteases inhibitors cocktail. After 30 minutes incubation on ice, the suspension was centrifuged at 14000 rpm for 30 minutes at 4°C and supernatant used for further analysis. Protein-DNA complexes were resolved by non-denaturing 5% polyacrylamide gel electrophoresis and specific STAT3/DNA complexes were detected by autoradiography. Poly(dI-dC) was added as competitor in a 500-, 1000- or 2000-fold excess. As control experiment EMSA analysis was carried out using a mutated probe #3 (TGCAGGAGAATCACTTCC**AT**G**TT**GGGGGAGGTTCA, where the mutated bases are indicated in bold characters).

### DNA-Affinity Assay

A biotinylated DNA fragments corresponding to the 5′-flanking region (−4379/−4229) of the human *Tpx2* gene was prepared by PCR using the following primers: 5′-CTAAAAAATTAGCTGGGCGTC-3′ and 5′-biotin-GACAAAATTTCGCTCTTTCAC-3′. This probe was immobilized on magnetic M-280-Streptavidin Dynabeads (Dynal, InVitrogen, Carlsbad, CA, USA) (200 pmol/mg beads) and incubated with nuclear extracts (2.5 mg protein/50 pmol probe) in the presence of a 1000-fold excess poly(dI-dC). After several wash steps specifically bound proteins were eluted with Laemmli buffer (62,5 mM Tris-HCl pH 6.8, 2% SDS, 10% v/v glycerol, 5 mM DTT, 0.02% bromophenol blue), resolved by SDS-gel electrophoresis in 10% polyacrylamide and analyzed by Western blotting using a polyclonal anti-STAT3 (Phospho-Tyr-705-STAT3) antibody (Cell Signaling Technology, Beverly, MA, USA). A control experiment was carried out using streptavidin beads saturated with biotin.

### Chromatin immunoprecipitation (ChIP)

Chromatin immunoprecipitation was performed essentially as previously described [14]. Briefly, DNA-protein cross-linked complexes were formed in M14 cells using cis-diamminedichloroplatinum. DNA-protein cross-linked complexes were purified by gel filtration and subjected to immunoprecipitation with a polyclonal anti-STAT3 antibody (Santa Cruz Biotechnologies, Santa Cruz, CA, USA). The DNA was purified and amplified by PCR using the same two primers mentioned above for the DNA-binding assay.

### *Tpx2* mRNA Determination

Tpx2 mRNA was determined by Quantitative Real Time Polymerase Chain Reaction (RT-PCR). M14 and ARO cells, treated without or with inhibitory peptide, were harvested (∼1×10^6^ cells) and total RNA was isolated using TRIzol (Invitrogen, Carlsbad, CA, USA). Aliquot of RNA (1 µg) was reverse transcripted by using Side-Step QRT-PCR cDNA Synthesis Kit (Stratagene).

RT-PCR was performed using specific primers for *Tpx2* and *GAPDH* (SuperArray Biosciences Corporation, Beverly, MA, USA). SYBR green-based (Brilliant_SYBR_Green QPCR Master Mix, Stratagene) RT-PCR was performed with a MJ Mini Opticon Detection System (BioRad Laboratories). The following protocol was used: denaturation step (95°C for 5 min), annealing (62°C for 30 sec) and elongation (72°C for 30 sec) steps repeated 40 times. All reactions were performed in triplicate. *GAPDH* gene was used for normalization. Quantification of *Tpx2* mRNA was obtained using the Gene Expression analysis tool of iCycler iQ Real-Time PCR detection system (Version 1.10, BioRad Laboratories).

### STAT3 Luciferase Reporter Assay

The DNA fragment corresponding to the 5′-flanking region (−4379/−4229) of the human *Tpx2* gene was generated by PCR amplification of human genomic DNA. The primers used in the PCR reaction were: *Tpx2*-sacI 5′-GGGAGCTCGTCTGTCTTTTTAC-3′ and *Tpx2*-bglII 5′-GGGAGATCTGAAATTTGTAAGGTAAC-3′. The 5′-flanking fragment was then cloned into the Sac/BglII site of firely luciferase pGL3-promoter vector (Promega) and verified by DNA sequencing. For luciferase assay, M14 cells (5×10^4^ cells/well) grown in a standard culture medium containing FBS were transiently transfected with the reporter plasmid and the pGL3-Control Vector as negative control, using the TurboFect Transfection Reagent (Fermentas) according to the manufacturer's instructions. After 24 hours, untreated and treated with inhibitory peptide cells were lysed, and luciferase activity was determined by a chemiluminescence assay using the luciferase assay kit (Promega) and Appliskan luminometer (Thermo Scientific).

### Proliferation and cell cycle analysis

M14 and ARO cells were seeded at a density of 200,000 cells/well in a 6-well plate under standard conditions. To analyze cell proliferation, cells were grown in RPMI-1640 medium with or without 1 mM STAT3 inhibitory peptide. After 24 h and 48 h of incubation, cells were examined by microscope (Leica AF6000 Modular Systems) with 20× objective and subsequently counted. To analyze the cell cycle, M14 and ARO cells were treated with 1 mM STAT3 inhibitory peptide for 24 h, trypsinized, washed twice with PBS and collected by centrifugation. The pellets were fixed in 70% ethanol at 4°C 24 h, washed two times with ice-cold PBS and resuspended in 500 µL PBS. Cell suspensions were incubated with RNase A (50 µg/mL) for 30 min at 37°C, sequentially stained with 100 µg/mL propidium iodide (PI) for 1 h and analyzed by BD Accuri C6 flow cytometer. At least three-independent experiments were performed.

### Immunofluorescence staining

Immunofluorescence analysis was done on the cultured cells according to the protocol described by Wittmann et al. [15]. Briefly, M14 and ARO cells were grown on glass coverslips, washed twice with phosphate-buffered saline (PBS) and fixed with cold methanol for 30 minutes. Subsequently, the cells were permeabilized with PBS containing 0,2% TritonX-100 (PBST) for 30 minutes and blocked with PBST containing 2% BSA (Serva). Immunostaining was performed using a TPX2 (D2R5C) XP monoclonal antibody (Cell Signaling Technology) (1∶500 diluted in PBST containing 1% BSA) for 1 hours. Following the washes with PBS, the cells were incubated for 1 h in the dark with a secondary antibody FITC-conjugated anti-rabbit IgG (Jackson Immunoresearch, West Grove, PA) (1∶200 diluted in PBST containing 1% BSA). The nuclei were finally counterstained with diamidinophenylindole 50 ng/ml for 1 minute. After washing with PBS, glass microscopy slides, mounted with Vectashield, were examined by microscope (Leica AF6000 Modular Systems) with 63× oil immersion objective.

## Results and Discussion

Among the genes whose expression is regulated by STAT3 and which may contribute to the oncogenic potential of STAT3 are those coding for the anti-apoptotic proteins BCL-2, MCL-1, SURVIVIN, and BCL-X_L_. However, the binding of STAT3 on the promoters of these genes has not always been experimentally demonstrated. In particular, the 5′-flanking region of the human *Bcl2l1* gene, coding for BCL-X_L_, has many potentially binding sites for STAT proteins, but none of these correspond closely to the consensus sequence of STAT3. An inspection of this region on human chromosome 20 has revealed that *Tpx2* gene is close to *Bcl2l1* and the two genes being transcribed in opposite direction on DNA, so that they have in common a 5′-flanking region of more than 16,000 base pairs (Figure 1A).

In order to ascertain the presence of high-affinity binding sites for STAT3 within this region, we carried out gel-retardation experiments using nuclear extracts known to contain activated STAT3 and DNA fragments containing the putative binding sequences. For this purpose, we used nuclear extracts from M14 melanoma cells, where STAT3 has already been shown to be constitutively activated [13]. Four potential binding sites for STAT3 have been examined, as shown in Figure 1A.

**Figure 2:**
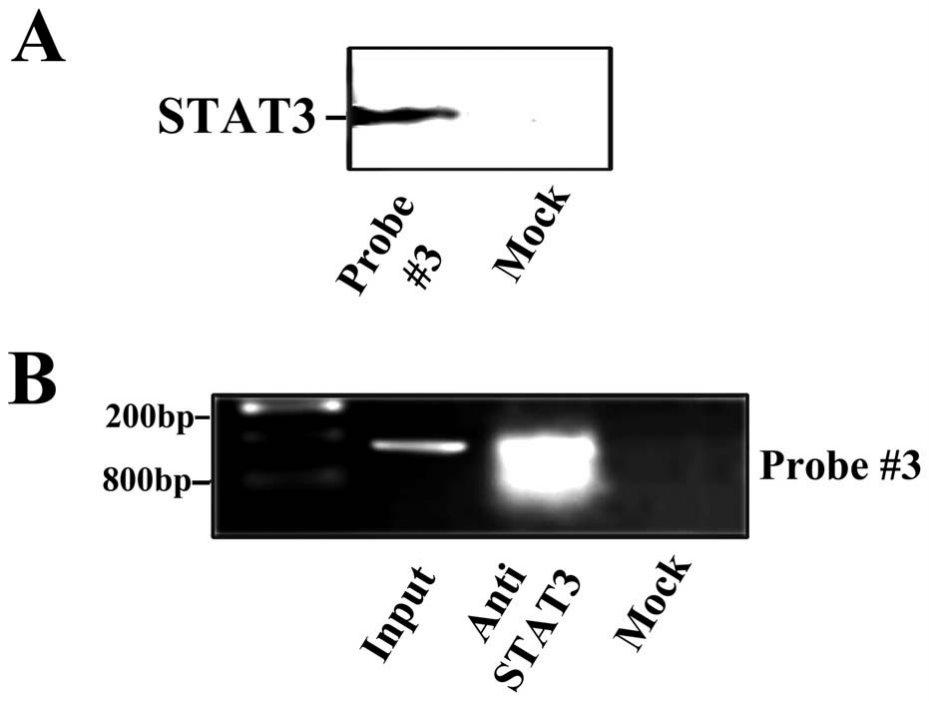
*In vitro* and *in vivo* binding of STAT3 to the DNA fragment #3. (**A**): Western blotting analysis using a polyclonal anti-STAT3 (Phospho-tyr-705-Stat3) antibody of bound proteins eluted from a DNA fragment corresponding to the 5′-flanking region (−4379/−4229) of the human *Tpx2*. A control experiment was carried out using streptavidin beads saturated with biotin. (**B**): PCR analysis to verify the enrichment of the specific STAT3-binding site present in the immunoprecipitated DNA in comparison with a mock immunoprecipitation (performed with preimmune IgGs). Input: genomic DNA (100 ng). Anti-STAT3: DNA (100 ng) immunoprecipitated with anti-STAT3 antibody. Mock: DNA (100 ng) from a mock immunoprecipitation.

Only one of the four potential binding sites examined appeared to bind STAT3 with high affinity, since the binding was not affected by the presence of a 1000-fold excess of competitor DNA (Figure 1B). This site is located at −4305/−4297 base pairs from the transcription start of *Tpx2* and has a -TTCCCGGAA- sequence, which is identical to the sequence bound by activated STAT3 in the promoter of gene *Cdkn1a*, coding for the protein P21^WAF1/CIP1^. No complexes were observed when the gel-retardation was performed with a similar DNA fragment, mutated within the STAT3 consensus sequence (Figure 1C). It is known, however, that the binding of the various STAT proteins to DNA cannot always be predicted *a priori* from their consensus sequences, so that other members of the STAT family might have been responsible for the formation of the DNA-protein complexes we observed. To identify the specific STAT protein involved in the observed interaction, a 150-base pairs DNA fragment (−4379 to −4229 in the 5′-flanking region of the *Tpx2* gene), containing the same consensus sequence, was immobilized on magnetic beads and tested for the binding of proteins from the M14-nuclear extract in the presence of a 1000-fold excess of aspecific DNA. The proteins that were specifically bound were eluted and analyzed by gel electrophoresis and Western blotting, and STAT3 was clearly identified among them, as shown in Figure 2A.

These results demonstrate that the examined region in the 5′-flanking sequence of *Tpx2* gene is able to bind STAT3 *in vitro* with high affinity. To detect the binding of STAT3 *in vivo* to the same sequence, a chromatin immunoprecipitation experiment was carried out on M14 cells. DNA- proteins cross-linking were performed by treating intact cells with cis-diamminedichloroplatinum. This cross-linking reagent was chosen for its high efficiency and for its capacity to identify proteins directly interacting with DNA, since, contrary to formaldehyde, it has no propensity to form protein-protein cross-linkages [16]. The DNA-STAT3 complexes were immunoprecipitated with an anti-STAT3 antibody and the immunopurified DNA was extracted and analyzed by PCR. As shown in Figure 2B, the immunoprecipitated DNA was enriched with the same 150-base pairs region used for the *in vitro* experiment, containing the STAT3 consensus sequence as described before.

**Figure 3:**
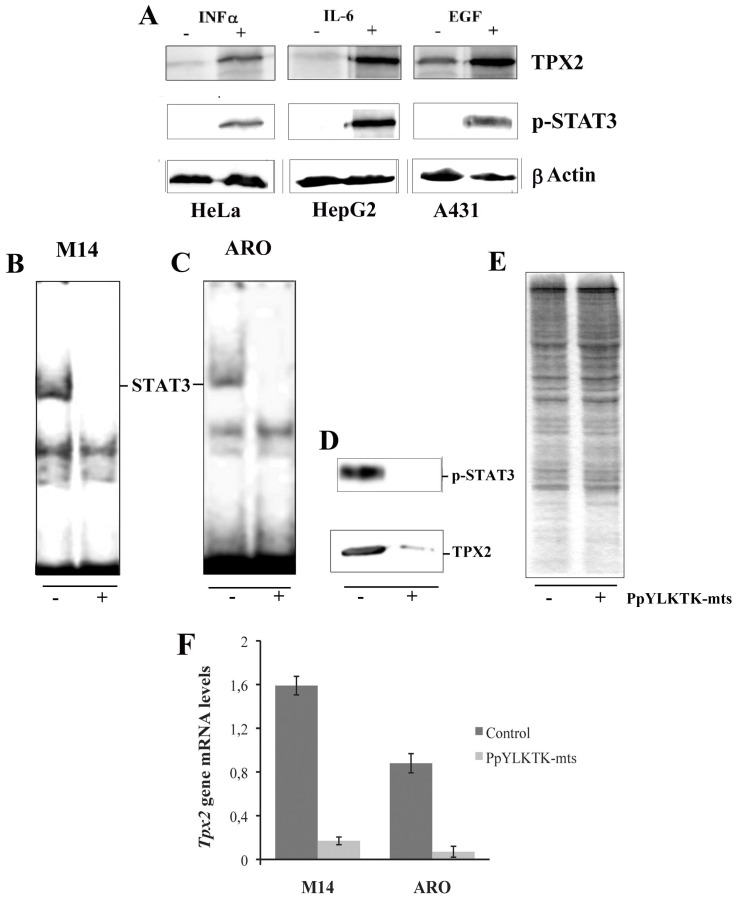
The correlation between p-STAT3 (Y705) and Tpx2 expression levels and effects of STAT3 inhibition by the PpYLKTK-mts inhibitory peptide in M14 and ARO cells. (**A**) Western blotting analysis of TPX2 in HeLa, HepG2 and A431 cell lines after specific activation of STAT3 pathways. (**B, C**): EMSA analysis with radiolabeled probe #3. Nuclear extracts from M14 (**B**) and ARO (**C**) cells were preincubated for 30 min without (−) or with (+) 1 mM inhibitory peptide before the addition to the probe. (**D**): Effect of the inhibitory peptide on p-STAT3 and TPX2 proteins in M14 cells. Nuclear extracts from M14 cells untreated (−) or treated (+) with the inhibitory peptide were resolved on SDS-PAGE and subjected to western blot analysis with Ab STAT3-Tyr^705^ and Ab TPX2 antibodies. (**E**) Gel was stained with Coomassie, to show that the same amount of proteins of nuclear extracts, from untreated or treated cells, were subjected to electrophoresis. (**F**): RT-PCR analysis of *Tpx2* gene expression normalized to *GAPDH* in M14 and ARO cells untreated (Control) or treated (PpYLKTK-mts) with inhibitory peptide.

Thus, constitutively activated STAT3 is bound *in vivo* to this site found in the 5′-region of the *Tpx2* gene. This finding supported the possibility that STAT3 is involved in the regulation of *Tpx2* expression. To verify this hypothesis, we tested on different cell lines (HeLa, HepG2 and A431) whether the specific activation of STAT3 could influence the expression of TPX2 protein. Western blotting images reported in Figure 3A, show a clear increase in the amount of the TPX2 protein in nuclear extracts where the STAT3 has been specifically activated.

The effect of a specific inhibitor of STAT3 on gene expression was also tested on M14 and ARO cell lines. Turkson et al. [11] have shown that a phosphopeptide able to interact with the SH2 site of STAT3 inhibits the binding of STAT3 to DNA *in vitro* and *in vivo*, probably by disrupting the active STAT3 dimers. When used *in vivo*, it might also associate with non-phosphorylated cytoplasmic STAT3 monomers and form STAT3-peptide complexes that would be incapable of binding to the docking sites of receptors for subsequent *de novo* phosphorylation and activation. By adding a membrane translocating sequence [12] the peptide acquires full cell-membrane permeability. First, this peptide was tested for its inhibitory activity on the formation of the STAT3-DNA complex as detected by gel retardation using a nuclear extracts of M14 cells and a DNA fragment containing the previously described consensus sequence. As expected, the presence of the inihibitory peptide in the nuclear extracts abolished the formation of the complex (Figure 3B). Next, cultured M14 cells were treated with the inihibitory peptide and then assayed for the presence of STAT3-Y705 phosphorylation and TPX2 by gel electrophoresis and Western blotting. Figure 3D shows that treatment with the inhibitory peptide decreased STAT3-Y705 phosphorylation and significantly reduced the amount of TPX2, while leaving essentially unaltered the total proteins of whole nuclear extracts as evidenced by Coomassie staining (Figure 3E). To confirm this result, the effect of the same peptide on another cell line was tested. We examined ARO cells, for the presence of constitutively activated STAT3, which was indeed found to be present as shown by gel-retardation (Figure 3C). When these cells were treated with the inhibitory peptide, the nuclear extracts were no more able to retard the STAT3 consensus sequence (Figure 3C). The effect of the inhibitory peptide on the transcription level of *Tpx2* gene was analyzed by RT-PCR performed on M14 and ARO cells. As shown in Figure 3F, *Tpx2* expression was strongly decreased by the action of the inhibitory peptide in comparison to the untreated cells.

**Figure 4:**
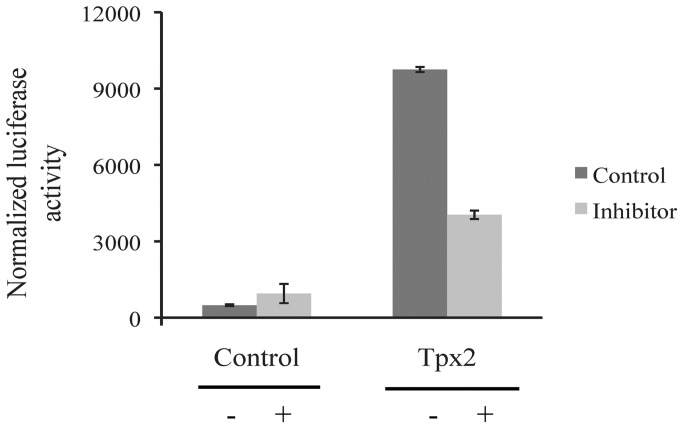
STAT3 regulates *Tpx2* gene expression. *Tpx2* promoter activity in M14 cells after treatment without (−) and with (+) inhibitory peptide. DNA fragment corresponding to 5′-flanking region (−4379/−4229) of the human *Tpx2* gene was cloned into the Sac/BglII site of firely luciferase pGL3-promoter vector (Promega). As negative control pGL3-control vector was used.

To demonstrate that STAT3 activation can regulate *in vivo Tpx2* expression, M14 cells were transiently transfected with a luciferase reporter construct containing the −4379 to −4229 region of the putative STAT3 binding site for a reporter activity assay, in the presence or in the absence of the inhibitory peptide. Untreated M14 cells showed an eightfold increase of luciferase expression, as result of the constitutively activated STAT3 in this cell line; this effect was repressed in M14 cells treated for 24 hours with the STAT3 inhibitor (Figure 4).

As a whole, these results indicate that STAT3 contributes to the activation of *Tpx2* expression. Its high-affinity binding site at −4305/−4297 bp from the transcription start is likely to be involved in this regulation. In fact, this site is bound *in vivo* by STAT3 and its sequence is identical to the well-characterized STAT3-binding site in the *Cdkn1a* gene. Furthermore an examination of the corresponding 5′-region of the mouse *Tpx2* gene on chromosome 2 revealed the existence of a STAT3 consensus sequence at −3965/−3957 base pairs. The sequence similarity in the same gene-flanking region of genoma of different species supports the existence of similar regulatory mechanisms and the intervention of the same transcription factors.

Regulatory binding sites for STAT proteins are often found at a short distance from the promoter. However, binding sites localized at higher distance has also been described. Thus, for example, a site for STAT3 at −1093 has been identified in the human *Perforin* gene [17], and for STAT5 at −4285 in the human *IL-2R*α gene [18].

Considering that the *Bcl2l1* and the *Tpx2* genes share the same 5′-region in common, it could be argued that the STAT3 site that we described might also regulate the expression of *Bcl2l1*. In fact the activation of STAT3 is often associated with an over-expression of the anti-apoptotic BCL-x protein. However, the possibility that this site is responsible for this regulation appears to be unlikely considering its distance (about 12 Kbp) from the *Bcl2l1* gene. Furthermore, an analysis carried out by the MAR-Finder program identified a region interposed between *Bcl2l1* and *Tpx2* sites having a strong character of a MAR (matrix associated region). It is generally held that such DNA regions, which have a tendency to associate with the nuclear matrix can act as insulating elements between independently regulated chromatin domains.

**Figure 5:**
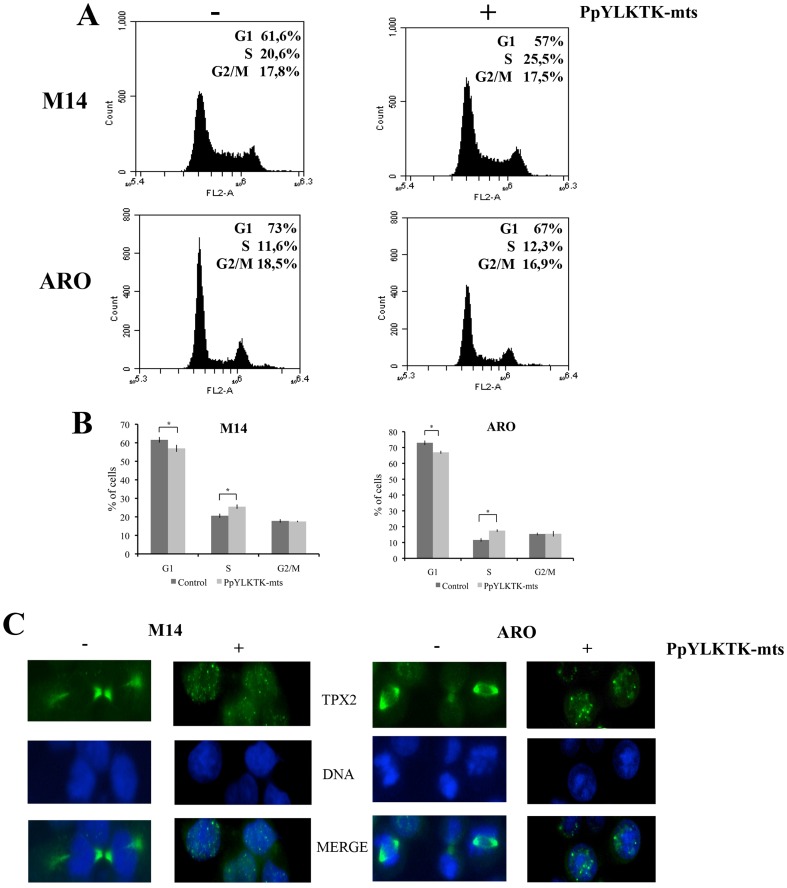
Regulation of mitosis by STAT3-TPX2 axis. (**A**) Cell cycle analyses by BD Accuri C6 flow cytometer. M14 and ARO cells were treated or untreated with STAT3 inhibitory peptide. Percentages of cells in the G1, S, or G2/M phases of the cell cycle are indicated. (**B**) Compilation of cell cycle analyses from three independent experiments. Standard deviations are indicated. Asterisks equal p<0.05. (C) Immunofluorescence images of TPX2 (green) and of DNA (Blu) in M14 and ARO cell lines without and with the inhibitory peptide. In the absence of inhibitor, TPX2 localize to the mitotic spindle, instead in the presence of the inhibitor is predominantly nuclear.

**Figure 6:**
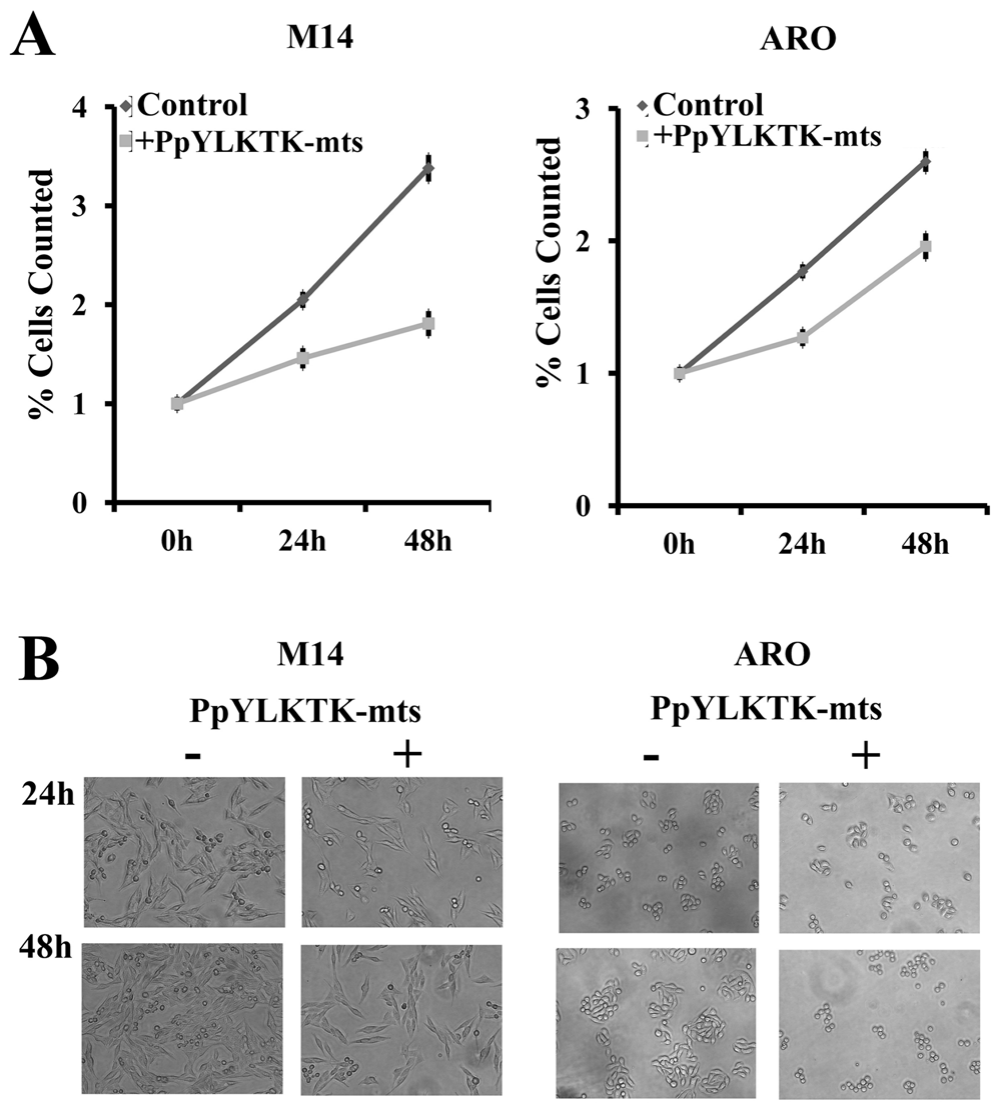
Inhibition of STAT3-TPX2 axis reduces cell proliferation of M14 and ARO cell lines. (**A**) Cell counts following to treatment of 24 h and 48 h with or without the inhibitory peptide. Graphs represent relative cell numbers, at the indicated time points after incubation with inhibitory peptide. Cell numbers seeded (time point 0) were set as 1.0. Experiments were performed in triplicates, standard deviations are indicated. (**B**) Representative cell proliferation images.

Since TPX2 is a microtubule associated protein necessary for mitosis, other activating transcription factors have to be present even when STAT3 is not activated. However, it appears that activated STAT3 produces an increase in the transcription level of the gene, contributing to the over-expression of TPX2 observed in a variety of tumours as hepatocellular carcinoma [19] lung [20], prostate [21], ovarian [22], pancreatic [23] and colon [24] cancer cell lines. Our hypothesis that the STAT3-TPX2 axis can regulate the progression and the cell mitotic process is clearly supported from the results of experiments by flow cytometry and immunofluorescence on M14 and ARO cells, all performed in the absence or presence of the STAT3 inhibitory peptide. Cell cycle analysis, performed by flow cytometric assay provided evidences that TPX2 downregulation by STAT3 pathway inhibition may result in cell cycle arrest, since inhibitory peptide treatment increased the percentage of S-phase cells (Figure 5A). Moreover, immunofluorescence studies demonstrate that in cells treated with the STAT3-specific inhibitory peptide, the lower expression of the protein TPX2 results in an alteration of the mitotic spindle formation (Figure 5B). Cell proliferation assay showed a decrease in cell number after treatment with the inhibitory peptide and it is possible that cell cycle arrest induced by STAT3 inhibition may contribute to this observation (Figure 6).

In summary, we provide evidence that activated STAT3 contributes to the overexpression of TPX2, through the binding to 5′-flanking region (−4379/−4229) of *Tpx2* promoter and that STAT3 -TPX2 pathway influences cell proliferation and mitosis.
